# Nurse Manager's Responsibilities in Creating Supportive Working Conditions Post Implementation of Everyday Coping: A Hermeneutic Research Study of District Nurses' Experiences

**DOI:** 10.1155/2024/1089785

**Published:** 2024-05-20

**Authors:** Marianne Hauan, Kari Kvigne, Johanne Alteren

**Affiliations:** ^1^Faculty of Nursing and Health Science, Nord University, Mo i Rana 8622, Norway; ^2^Faculty of Health and Social Science, Inland University of Applied Science, Elverum 2418, Norway; ^3^Faculty of Health Science and Social Care, Molde University College Specialized University in Logistics, Molde 6410, Norway

## Abstract

**Aim:**

To gain knowledge about how district nurses experience changes in working conditions and discuss nursing manager's responsibility in facilitating working conditions for district nurses following the implementation of everyday coping.

**Background:**

To overcome the challenges related to the sustainability of the healthcare sector, everyday coping was implemented in district nursing. The implementation was set by the government and implemented by the municipality. The nursing manager has an overall responsibility to facilitate working conditions so that everyday coping can be applied into district nursing practice.

**Method:**

This descriptive and interpretative study involved 19 interviews and 19 observations on 10 nurses. Kvale and Brinkmann's three levels of understanding were employed in the analysis.

**Results:**

Three categories were established based on the results of the data analyses: (i) time and space are not considered, (ii) crossfire of conflicting expectations, and (iii) nursing manager's commitment to everyday coping.

**Conclusion:**

The working conditions of district nurses are not adapted for them to work according to the everyday coping mindset. The nursing manager has a key role in supporting nurses and addressing challenges the nurses meet in their daily work, where everyday coping is implemented. The study highlights the importance for healthcare managers, at various levels in the healthcare sector, to be conscious of the district nursing practice, its complexity, and quality of health services when implementing change. This knowledge is important when planning future healthcare and nursing practice.

## 1. Background

Globally, politically entailed strategies are introduced and implemented into healthcare to meet the changing needs of the ageing population, with the goal of achieving sustainable healthcare services [1–4]. In Norway, one of these political strategies is the implementation of the Norwegian-developed mindset of everyday coping (Norwegian: Hverdagsmestring) in the practice of district nursing [1, 5]. Everyday coping is a health-promoting and rehabilitative mindset that emphasises the individual's coping in everyday life regardless of physical functional level [6, 7]. This mindset involves guiding, educating, facilitating, and motivating patients to become more self-conscious, increase independence, and maintain self-care [1, 6–12].

Everyday coping is a mindset developed by healthcare professionals, mainly physiotherapists and occupational therapists [6]. It has been used as a politically initiated strategy for providing healthcare services nationally [1] and was implemented into district nursing in several municipalities in Norway. The fact that it is politicians who are the initiators of the everyday coping mindset being implemented in district nursing services in Norway and not the nurses themselves indicates a top-down approach was used to adopt everyday coping in district nursing practice. The top-down implementation approach has been criticised as it does not promote willingness and commitment to sustain the change because the nurses who work in the healthcare service are not involved in the implementation process [13]. According to Harrison et al. [14], those who are affected by change are more likely to commit to change if they are part of the implementation process.

To implement change in an organisation, change management is key, which is an important part of the nursing manager's responsibility [15]. Change management involves planning, analysing, engaging, thinking, and doing to successfully execute a strategy that achieves sustainable results in a practice [15]. However, Shanley [13] highlighted that change is a complex process that occurs over time and is affected by several unpredictable variables.

A study by Hauan et al. [16] showed that district nurses provide care to patients based on their values and professional knowledge and the situations of individual patients. Wittrock et al. [5] investigated the implementation process of everyday coping in two municipalities in Norway. They showed that district nurses experience limitations in their working conditions, such as increased time pressure when working according to the everyday coping mindset in patient situations [5]. A previous Swedish study showed that the overall work situation of home care workers was worse in 2015 than in 2005. The deterioration in working conditions was due to the increasing number of patients admitted each day, lack of support from the manager, and limited time to discuss difficult situations with coworkers [17]. Strandell [17] also emphasised that the deteriorating working conditions may be related to cutbacks and organisational changes. Likewise, Shanley [13] emphasised that changes in the organisation of the public sector are often driven by the cost-cutting and rationalisation of services.

Previous research has shown that the implementation of change in practice, such as everyday coping in district nursing, has a significant impact on district nurses' working conditions. However, there is limited research on district nursing experiences following the implementation of the everyday coping mindset. Additionally, there is limited research on whether working conditions are sufficiently facilitated for nurses to be able to work according to the everyday coping mindset. Therefore, this study makes a novel contribution to the literature by gaining knowledge from a district nursing perspective about how district nurses experience changes in working conditions and discussing nursing manager's responsibilities in facilitating working conditions for district nurses following the implementation of everyday coping. This knowledge is of critical importance for nursing managers, who are required to implement similar health-promoting and rehabilitative mindsets or strategies in nursing services. Furthermore, the knowledge from this study is relevant for managers at various levels in the health and care sector as well as politicians.

## 2. Methods

### 2.1. Study Design

A descriptive and interpretive study using a hermeneutic approach was conducted [18]. The Standards for Reporting Qualitative Research guided the reporting of this study [19].

### 2.2. Overview of District Nursing in the Studied Municipality

The studied municipality has 26,000 inhabitants and a geographical area of 4,400 kilometres [20].

District nursing in Norway ensures that an individual patient receives the services they need at the right time. It provides a comprehensive, coordinated, and flexible service that ensures continuity. This service is provided to young children, adults, and older people. The provision of district nursing services has no time limits; hence, the time period of a patient receiving healthcare can vary from one single visit to several years [21].

When an individual needs healthcare assistance, the allocation office in the municipality has first contact with this person. The allocation office's employees are primarily nurses who perform home visits, in agreement with the patients, to determine the type of nursing care that the patient requires. A written contract, i.e., resolution, includes the type of nursing care the patient will receive, e.g., “assistance in performing personal hygiene or assistance in administering medications,” and contains the date when the content of the resolution will be reviewed, e.g., “within 6 months.” The resolution is then sent to the district nursing service, and the nursing manager will follow up the patient's healthcare needs described in the resolution.

The nursing manager of the district nursing service is a registered nurse and is responsible for managing the district nurses and the daily operation of this service. The nursing manager has the professional and administrative responsibility in the workplace. This means, among other things, responsibility for ensuring that the patients receive the help they need, assessed from a professional point of view, as well as facilitating the working conditions so that the help for the patients can be obtained from the district nurses. The nursing manager of the district nursing service operates under the supervision of the municipality's head of healthcare services. Furthermore, in this article, the manager of the district nursing service will be referred to as the nursing manager.

District nurses work in two shifts—morning and evening. At the start of every shift, they receive a worklist that has been prepared by the nursing manager. The worklist is task-oriented and describes which assignments, based on the resolutions, should be performed on a certain patient, at what time, and for how long.

### 2.3. Implementation of Everyday Coping in District Nursing

The process of implementing everyday coping was based on the municipalities' strategy and direction document for 2014: the healthcare personnel shall be educated on working according to the everyday coping mindset, which means encouraging an attitude change towards rehabilitation and health promotion where patient independence and self-care are a priority.

The nursing manager and the reablement team, two physiotherapists, an occupational therapist, and a nurse, were responsible for organising the implementation of everyday coping into district nursing services. The reablement team was responsible for conducting lectures and providing information brochures and held biweekly meetings with the district nurses. The lectures contained information on the benefits of working with a mindset of everyday coping, both for patients and society. For example, increased physical activity levels reduce the impairment of physical functions and increase the level of self-care [1]. The lectures also consisted of examples of how to work according to the everyday coping mindset, focusing on performing everyday physical tasks. The biweekly meeting, held once every other week, was organised by the nursing manager, but involved professional discussions between district nurses and the reablement team regarding working with the mindsets of everyday coping in specific patient situations.

The nursing manager's responsibility included arranging the biweekly meetings and performing daily follow-up of everyday coping status in district nursing, such as conducting professional discussions with the district nurses. The nursing manager also communicated with other healthcare units, such as the allocation office if patients' resolutions needed to be changed or updated to meet the required care need.

### 2.4. Participants and Recruitment

District nurses were purposely selected from a municipality in Norway where the everyday coping mindset was implemented in the service. The researcher initially contacted the nursing manager of the district nurses, both verbally and in writing. The nursing manager informed 20 district nurses employed about the study's purpose and procedure, distributed the written information, discussed the relevant ethical issues, and asked for volunteers. Inclusion criteria for participation required nurses to have completed lectures on everyday coping to ensure familiarity and introduction to working according to the everyday coping mindset. Additionally, permanent position at the home care unit was a criterion, given the data collection period exceeding 6 months, thereby potentially increasing the risk of temporary staff being unavailable for the second round of data collection. Consequently, nurses who had not attended lectures on everyday coping and those without permanent positions were excluded from the study. Fourteen district nurses agreed to participate. Four of the nurses who did not have a permanent position at the home care unit were excluded. As for six district nurses, the reason for their refusal to participate remained unknown. Hence, ten district nurses, eight women, and two men with 10–18 years of experience participated in the study.

### 2.5. Data Collection

Data were collected in the following periods: September–October 2016 and February–March 2017. All district nurses were observed and interviewed in both periods, except for one district nurse who only participated in the last period. Thus, data collection involved 19 observations and 19 interviews.

#### 2.5.1. Observations

The observations were aimed at exploring the district nursing practice and their working conditions following the implementation of everyday coping, for example, how they prioritised the assignments on the worklists, collaborated with each other, the reablement team, and the nursing manager, and interacted with their patients.

#### 2.5.2. Interviews

The interviews were conducted in the form of a dialogue [18], which lasted for 18–65 minutes (mean: 36.2 minutes). The length of the interviews varied as some district nurses provided a more detailed answer, whereas others opted to provide a more concise response. The dialogues involved a discussion on how the district nurses experience their working conditions following the implementation of everyday coping, their collaboration with the reablement team, the nursing manager, and how the working conditions affected their ability to perform everyday coping. The interviews were digitally recorded, transcribed verbatim, and anonymised.

### 2.6. Data Analyses

The analysis was carried out using Kvale and Brinkmann's three levels of interpretation: self-understanding, critical understanding based on common sense, and theoretical understanding [18]. In the first level, self-understanding, we formulated what the district nurses themselves perceived as the meaning of their statements, that is, meaning-bearing units. Then, the meaning-bearing units were condensed and expressed similarly to the district nurses' self-understanding. In the second level, critical understanding based on common sense, the results were interpreted in a wider sense of understanding than the nurses' own understanding. Based on common sense and general knowledge of the statement's content, it is possible to clarify and enrich the interpretation of the statement. The condensed statements and observations with common characteristics were combined. The categories and concepts were revised as categories, which served as the basis for presenting the results (Table 1).

In the third level of interpretation, theoretical understanding, theory, and previous research were used to interpret the findings. The interpretation went further than the nurses' self-understanding and also further than the interpretation based on common sense. The third level is presented in the Discussion.

### 2.7. Ethical Considerations

The study was conducted in accordance with Norwegian law and the Declaration of Helsinki. The study was approved by the Regional Committees for Medical and Health Research Ethics (project number: 2015/2276 REK nord) and Norwegian Centre for Research Data (project number: 473228). The district nurses and patients received verbal and written information describing the study and signed informed consent. Personal information of patients participating in the observations was not collected. The district nurses and patients were informed about their option to withdraw from the study at any time without any consequences. The anonymity of the participants was ensured.

## 3. Results

Based on the results of the analysis, three categories emerged regarding the district nurses' working conditions following the implementation of everyday coping: (i) time and space are not considered, (ii) crossfire of conflicting expectations, and (iii) nursing managers' commitment to everyday coping.

### 3.1. Time and Space Are Not Considered

Effectively prioritising time was something the district nurses highlighted as an important part of their daily work. However, the time and space required to be able to work according to the everyday coping mindset were not considered by the nursing manager. The nurses emphasised time and space as a challenge in performing everyday coping.

The worklists served as a guide for the district nurses' workday. When district nurses are instructed to work according to the everyday coping mindset, it creates an expectation that they should facilitate tasks and motivate patients to be active, requiring time, space, and continuity to achieve independence and an increased level of self-care. The district nurses reported that the time and space required to work according to the everyday coping mindset were not present in the district nurses' worklists. They also expressed that in cases where time was limited, they provided a compensatory level of care, which was also indicated in the observations. If the time between assignments on the worklist was limited, the district nurses ensured that the patients received the help they needed rather than ensuring that the task was performed according to the everyday coping mindset.

“When we're in a hurry it's very easy to take the key from the key box instead of waiting for the patient to go to the door and open it.”

District nurses pointed out that it was difficult to stand beside the patient and allow them to independently perform the task, especially when another patient was waiting for their assistance. If the district nurses had other responsibilities to attend to, they preferred to perform the tasks on the patients' behalf. For example, the district nurses brought water to be taken with the medicines, turned on the lights in dark rooms, and removed the garbage, instead of asking the patient to perform these tasks with them. When the district nurses were under time pressure, they avoided situations that consumed the majority of their time guiding or motivating patients to perform certain activities.

“I think we do a lot for the patients that they can do themselves. I feel that every day. I think many times we do it because it is faster.”

To function according to the everyday coping mindset, the district nurses needed more time and space with the patients than the worklists indicated. This in turn leads to the nurses having little leeway to prioritise their time. The district nurses emphasised that several factors should be taken into account when prioritising the use of their time; for example, some patients experience difficulties in performing routine tasks and require a longer explanation, which should be taken into account when prioritising the time available on that day.

### 3.2. Crossfire of Conflicting Expectations

The district nurses described a feeling of being in a crossfire of conflicting expectations between the patients' request for help, nursing manager's requirement to work according to the everyday coping mindset, and outdated resolutions. According to the nurses, different expectations entail challenges in meeting the nursing manager's requirements.

One of the challenges reported is the lack of interdepartmental cooperation between the hospital and district nurses. An example is when patients leave the hospital. The patients are informed by the hospital that the district nurses will provide assistance to address their needs, which may create a breach of expectations regarding the mindset's requirements, where a patient's independence and ability to perform everyday activities are desired and encouraged.

“Patients are informed by the hospital that district nurses will help them with whatever they need. But it is supposed to be all about promoting independence. Yet, the patients end up expecting the district nurses to handle everything. It's like we and the hospital aren't quite on the same page.”

Another challenge is when the resolution that legislates tasks that the patients are entitled to was not in line with how the district nurses are instructed to perform their task according to the everyday coping mindset. Moreover, several district nurses claimed that the resolutions were not always in accordance with the patient's need and type of assistance requested because the resolution is not regularly updated based on the patient's change in function and their need for help. Hence, it remained a challenge for district nurses to ask the patients to perform parts of the tasks that they already had a resolution on. The district nurses described this scenario based on their experience with patients expressing anger if they did not help them with the task described in their resolution. By providing essential information and effectively communicating with the patients, some of them have become more motivated to contribute to performing their tasks.

“I have experienced that some patients have been upset because we have not done something we used to do before. However, once you have explained why, for some patients it is fine. It is a matter of communicating with them.”

However, some district nurses reported that this scenario was not the same for everyone. They found it challenging when the resolutions, patients, and nursing managers' expectations contradicted with what was required of them.

### 3.3. Nursing Manager's Commitment to Everyday Coping

The district nurses highlighted the nursing manager's engagement as key to maintaining focus on implementing the mindset of everyday coping in their daily work.

“It's also about the head nurse. It is important that the head nurse shares the same mindset as us who are out in the field.”

Several of the district nurses experienced that the nursing manager included everyday coping in professional discussions and as topics in meetings with the district nurses. For example, one of the district nurses pointed out a specific patient situation during the meeting; the district nurse had observed that a patient was fully capable of making his own dinner, which changed the description of the patient's need for help in the worklist from “prepare dinner for the patient” to “assist the patient in making dinner.” However, the patient's involvement in the decision was not mentioned by the nurse. The district nurses, in collaboration with the nursing manager, are responsible for documenting and informing the allocation office regarding the need for change in the resolution.

To follow up the implementation status of everyday coping, the district nurses described the biweekly meetings as a reminder to continue maintaining focus. They were concerned that the focus on implementing everyday coping would eventually fade if the biweekly meetings were discontinued. Several nurses expressed that the nursing manager's focus during the meetings created a good and constructive platform for discussing how they could work according to the everyday coping mindset.

“The head nurse attends the meetings on everyday coping with the reablement team, and she take notes. So, we have a plan around the patients that the head nurse also follows up.”

Another concern that the nurses raised was that the reablement team did not always have complete insight into the diversity of the patients in the unit, making it impossible to working according to the everyday coping mindset of the patients' situations. Several of the district nurses reported that the individual patient situation was decisive for how nurses choose to provide care; therefore, everyday coping should not be implemented in all patients' scenarios.

## 4. Discussion

The nursing manager of the district nursing services plays a crucial role in facilitating the working conditions of district nurses, especially when implementing changes such as everyday coping in district nursing. One key responsibility of managers in change management is to adapt the service working conditions for nurses to handle and adjust to change when delivering high-quality care to their patients. Nevertheless, our findings show that district nurses' working conditions are not sufficiently facilitated for the nurses to be able to work according to the everyday coping mindset in patient situations. In this study's third level of interpretation, theoretical understanding, we will interpret our findings with existing theory and research to discuss nursing managers' responsibility in facilitating nurses' working conditions in relation to implementation of the everyday coping mindset.

To implement the everyday coping mindset into district nursing, a great deal of responsibility was given to the nursing manager. This was attempted by having biweekly meetings with the reablement team, in addition to an engaged and committed nursing manager, who included the district nurses in professional discussions. The district nurses found this to be very important and decisive for maintaining focus on everyday coping when practising district nursing. A committed manager who meets the staff regularly is also shown by Fläckman et al. [22] as an important factor for nurses when undergoing change. This finding is further supported by Strandell [17], who stated that a lack of support from the manager may lead to deteriorated working conditions for home care workers. Although the nursing manager was engaged and committed in our study, the district nurses still faced several challenges related to their working conditions.

The lack of time and space to work according to the everyday coping mindset was highlighted as a challenge in the district nurses' working conditions. When district nurses had limited time, working according to the everyday coping mindset was de-prioritised, and a more hands-on type of care was chosen. The contradictions between the time-pressured worklists and the time and space required of the mindset remained a challenge to district nurses in performing their daily work. Hjelle et al. [23] stated that having sufficient time to apply professional knowledge when supervising and supporting older persons in everyday activities is significant when helping patients improve their ability to perform daily activities. When it comes to arranging daily working conditions for the district nurses, the nursing manager has a central responsibility, since it is the nursing manager who has the overall responsibility for change management in the service [15]. Previous research on nurses' experiences with change management shows that nurses stated that managers made some mistakes during the implementation process, such as lack of planning, where changes are made in a hurry [24]. Insufficiently adapted worklists may cause district nurses to be uninspired to continue working according to the everyday coping mindset.

Our findings show that nurses are unable to work according to the everyday coping mindset continuously if their working conditions are not sufficiently facilitated. Therefore, everyday coping as a political strategy to prevent a reduction in physical function, improve health, promote independence in performing daily activities, and limit the use of resources, economic resources, and healthcare personnel [1, 5, 7, 8] was unsuccessful. This may be a consequence of the top-down implementation process of everyday coping, in which district nurses are not involved in the implementation process, and therefore, the complexity of the district nursing context and their working conditions may not be taken into consideration [13, 25]. Davidson [15] highlighted that political actors often misunderstand the complexity of the healthcare system and have reorganised the services to improve power and structure rather than enhance the care and quality. Therefore, the nursing manager has a particular responsibility and opportunity to include nurses' opinions and experiences in the implementation process, to better adapt nurses' working conditions to make it possible to work according to the everyday coping mindset. Harrison et al. [14] reported that those directly or indirectly affected by change are more likely to commit to change when they are involved in making decisions and professional contributions to change, which can improve patient and staff experiences. Therefore, the nursing manager, who has daily connections with the district nurses, should include them in the planning and implementing of changes in nursing practice, such as new mindsets or strategies.

Furthermore, this study shows that nurses find it challenging when resolutions and patients' expectations contradict with the mindset of everyday coping that they are instructed to incorporate into their daily work. For example, patients express anger if the district nurse does not help with their task. The distinction between “doing for” and “letting the patients do as much as possible by themselves” may be related to the fact that a huge part of nursing is “hands-on” and “take part in” type of work. Therefore, it is not usual nor justifiable to work with a “hands-off” type of attitude in nursing, which is essential to consider when implementing everyday coping into nursing practice [10, 26]. The same trend is shown in Hauan et al. research [16], which showed that nurses do not work according to everyday coping with all patients. The district nurses always consider the individual patient's situation before deciding how to perform professional nursing for every patient [16]. The nursing manager has a responsibility to address these conflicts to ensure that patient care is not compromised. This may be done by encouraging open communication and promoting a culture of collaboration. To ensure that district nurses can manage situations where there are conflicting expectations, it is crucial for nursing managers to provide them with training and support. This will not only help the district nurses address these types of conflicts, but also provide emotional and psychological support to help district nurses with any stress or anxiety associated with their working conditions. Shanley [13] stated that management literature does not sufficiently consider the personal and emotional aspects of change management, thereby causing stress, low morale, disorientation, mistrust, and lack of commitment among staff.

Although the theory of everyday coping states that the mindset should suit everyone [7], our findings and Hauan et al. research [16] show that everyday coping does not suit all patients who receive district nursing. It is essential that the workplace culture and district nurses' working conditions facilitate discussions about which patients the mindset suits and who it does not, so that patients receive the right care. The nursing manager has the overall responsibility to ensure that patients receive the right care when they need it. The discussion about to whom the mindset should be prioritised (the professional discussion) and how this should be facilitated in the service (the administrative discussion) should also take place at a management level, among other managers in nursing services and with the municipality's head of healthcare services. Additionally, these discussions should be based on the experiences of district nurses and patients. It is surprising that the mindset about everyday coping in theory has a strong focus on user participation; however, from our findings, it is not clear how patients are involved in the decision-making process regarding their resolution that constitutes their right to nursing services.

More research is needed to better understand the mindset of everyday coping within a nursing practice, and the consequences that the everyday coping mindset has for patients receiving nursing services.

### 4.1. Strengths and limitations

In this qualitative study, efforts have been made to ensure trustworthiness by providing a sufficient description of the district nurses' working conditions following the implementation of everyday coping. Two methods have been used in the data collection: interviews and observations. These methods complement each other, as the district nurses in the interviews discussed and elaborated their practice more than observations or interviews alone can manage. During the interviews, the researcher summarised the essence of the conversation to ensure that the content was correctly understood [27]. The interviews contained open questions that provided the district nurses the opportunity to share how they experience practising professional nursing following the implementation of everyday coping [18]. All researchers participated in the analysis process, which strengthened the validity of the analysis. A limitation might be that no data were collected from the nursing managers, only from the district nurses' perspective on the nursing manager. Furthermore, studies could explore nursing managers' experiences with change management and implementation of the everyday coping mindset.

## 5. Conclusions

Our study illustrates the importance of management when implementing changes in nursing practice, such as everyday coping. The nursing manager must provide nurses with facilitated working conditions for the nurses to be able to work according to the everyday coping mindset. This includes addressing challenges such as lack of time and space and promoting communications and a culture of collaboration. Nursing manager's intermediary role allows them to communicate challenges and needs effectively upward to a higher organisational level within the healthcare system and to local politicians, addressing challenges such as time constraints and conflicting expectations.

Moreover, the utilisation of the everyday coping mindset is not universally applicable across all patient scenarios. In this context, the nursing manager could facilitate discussions concerning the possibilities of the everyday coping mindset, involving an examination of both feasible and less feasible patient demographics.

In addition, the nursing manager should include the district nurses in the implementation process and ensure district nurses are emotionally and psychologically prepared for the implementation of everyday coping. The nurse manager can actively involve district nurses in decision-making processes and guide their contributions. Ultimately, the nursing manager bear the overarching responsibility for the home care provided, highlighting the importance of their proactive engagement in ensuring conducive working conditions and quality of care.

## Figures and Tables

**Table 1:** Example of the analysis process, first and second levels of abstraction.

*Example of a meaning unit*: The duration of work according to the everyday coping mindset is not taken into account, especially when only three district nurses are available, and they are responsible for providing the healthcare needs of all the patients of an entire city
*Example of common-sense understanding*: District nurses describe their daily workload. Space and time are not taken into account when working according to the everyday coping mindset
*Example of a category*: Time and space are not considered

## Data Availability

The datasets generated and/or analysed during the current study are not publicly available due to permission, which has not been applied for from neither the participants nor due to Norwegian privacy legislation and the form signed by the participants about the study's privacy. The data generated are available from the corresponding author upon reasonable request.

## References

[1] Ministry of Health and Care Services (2018). *Leve Hele Livet- En Kvalitetsreform for Eldre [Live Your Whole Life- A Quality Reform for the Elderly]*.

[2] Ministry of Health and Care Services (2015). *Fremtidens Primærhelsetjeneste- Nærhet Og Helhet [The Primary Health and Care Services of Tomorrow– Localised and Integrated]*.

[3] Ministry of Health and Care Services (2013). *Morgendagens Omsorg [Future Care]*.

[4] World Health Organization (2022). *Ageing and Health*.

[5] Wittrock C., Ingelsrud M. H., Norvoll R. Ledelse og organisering av innovasjonsprosesser i Gran og Lunner kommuner. En casestudie av implementering av hverdagsmestring i helse og omsorg Management and organization of innovation processes in Gran and Lunner municipalities. *A Case Study of the Implementation of Everyday Coping in Health and Care*.

[6] Ness N. E., Laberg T., Haneborg M. (2012). *Hverdagsmestring Og Hverdagsrehabilitering [Everyday Coping and Reablement]*.

[7] Tuntland H., Hverdagsrehabilitering N. E. N. (2014).

[8] Aspinal F., Glasby J., Rostgaard T., Tuntland H., Westendorp R. G. (2016). New horizons: reablement- supporting older people towards independence. *Age and Ageing*.

[9] Hartviksen T. A., Sjølie B. M. (2017). *Hverdagsrehabilitering- Kvalitetsforbedring I Norske Kommuner [Reablement- Quality Improvement in Norwegian Municipalities]*.

[10] Ness N. E. (2016). Hverdagsmestring” [everyday coping]. *Ergoterapeuten*.

[11] Tinetti M. E., Charpentier P., Gottschalk M., Baker D. I. (2012). Effect of a restorative model of posthospital home care on hospital readmissions. *Journal of the American Geriatrics Society*.

[12] Langeland E., Tuntland H., Folkestad B., Førland O., Jacobsen F. F., Kjeken I. (2019). A multicenter investigation of reablement in Norway: a clinical controlled trial. *BMC Geriatrics*.

[13] Shanley C. (2007). Management of change for nurses: lessons from the discipline of organizational studies. *Journal of Nursing Management*.

[14] Harrison R., Fischer S., Walpola R. L. (2021). Where do models for change management, improvement and implementation meet? A systematic review of the applications of change management models in healthcare. *Journal of Healthcare Leadership*.

[15] Davidson J. (2015). What’s all the buzz about change management?. *Health Management Forum*.

[16] Hauan M., Kvigne K., Alteren J. (2023). Politically engaged mindset of everyday coping in relation to nursing values: a phenomenological-hermeneutic study of district nurses’ experiences. *SAGE Open Nursing*.

[17] Strandell R. (2020). Care workers under pressure- A comparison of the work situation in Swedish home care 2005 and 2015. *Health and Social Care in the Community*.

[18] Brinkmann S., Kvale S. (2015). *InterViews: Learning the Craft of Qualitative Research Interviewing*.

[19] O’Brien B. C., Harris I. B., Beckman T. J., Reed D. A., Cook D. A. (2014). Standards for reporting qualitative research: a synthesis of recommendations. *Academic Medicine*.

[20] Statistics Norway (2020). *Municipality Facts (The Municipality Is Anonymised in This Reference)*.

[21] Helse Norge Home nursing and other healthcare services in the home 2019.

[22] Fläckman B., Hansebo G., Kihlgren A. (2009). Struggling to adapt: caring for older persons while under threat of organizational change and termination notice. *Nursing Inquiry*.

[23] Hjelle K. M., Skutle O., Førland O., Alvsvåg H. (2016). The reablement team rsquo;s voice: a qualitative study of how an integrated multidisciplinary team experiences participation in reablement. *Journal of Multidisciplinary Healthcare*.

[24] Celebi Cakiroglu O., Ulutas Hobek G., Harmanci Seren A. K. (2022). Nurses’ views on change management in health care settings: a qualitative study. *Journal of Nursing Management*.

[25] Nilsen P. (2015). Making sense of implementation theories, models and frameworks. *Implementation Science*.

[26] Hauan M., Kvigne K., Alteren J. (2021). Fremtidens helse- og omsorgstjenester fra et hverdagsmestringsperspektiv [The health and care services of the future from an everyday coping perspective]. *Klinisk Sygepleje*.

[27] Sandelowski M., Barroso J. (2002). Finding the findings in qualitative studies. *Journal of Nursing Scholarship*.

